# Evaluation of Silodosin and Pelvic Floor Muscle Training in Men with Benign Prostatic Hyperplasia and Overactive Bladder (Silodosing) Study Protocol (Spirit Compliant)

**DOI:** 10.3390/ijerph182111426

**Published:** 2021-10-30

**Authors:** Magdalena Hagovska, Jan Svihra

**Affiliations:** 1 Department of Physiatry, Balneology, and Medical Rehabilitation, Faculty of Medicine, PJ Safarik University, 040 01 Kosice, Slovakia; magdalena.hagovska@upjs.sk; 2 Department of Urology, Jessenius Faculty of Medicine, Martin, Comenius University, 814 99 Bratislava, Slovakia

**Keywords:** silodosin, pelvic floor exercise, overactive bladder

## Abstract

The aim of our study will be to evaluate the effect of combining pelvic floor muscle training (PFMT) with the urgency-suppression technique and silodosin in comparison with silodosin alone in men with Benign Prostatic Hyperplasia (BPH) and Overactive Bladder (OAB) after 12 weeks of treatment. The primary outcome will be a change in the number of voidings and intensity of urgencies over 24 h using a micturition diary, and the secondary outcomes will be a change in lower urinary tract symptoms, a change in incontinence quality of life, a change in patients’ global impression of improvement, and a lower incidence of adverse events. A randomized intervention parallel multicenter study will be conducted in collaboration with 45 urological clinics at the national level. Patients will be assigned at a 1:1 ratio to the experimental and control groups using simple randomization according to odd and even patient sequence numbers in each ambulatory clinic. The experimental group will receive oral silodosin at a daily dose of 8 mg once daily and pelvic floor muscle training (PFMT) 5 times a week for 20–30 min a day, for 12 weeks. The control group will receive oral treatment with silodosin at a daily dose of 8 mg once daily for 12 weeks. The study protocol presents the starting points and design of a randomized, interventional, parallel, multicenter study looking at the effect of a combination of silodosin and PFMT versus silodosin treatment in men with BPH and OAB.

## 1. Introduction

The International Society for Continence (ICS) and the International Urogynecological Society (IUGA) define overactive bladder (OAB) as urgency with frequent urination and nocturia with or without urgent incontinence in the absence of a urinary tract infection or other disease [1]. All of these symptoms contribute significantly to the deterioration of quality of life for both sexes. An EPIC study estimated the prevalence of lower urinary tract symptoms (LUTS) in men and women using ICS definitions. The prevalence of male urinary storage symptoms (51%) was higher than the prevalence of urinary emptying symptoms (25%) and post micturition symptoms (16%). The overall prevalence of OAB was 11.8% in both sexes, and its frequency increased with age [2]. Lower urinary tract symptoms (LUTS) due to benign prostatic hyperplasia (BPH) are common in aging men and cause a deterioration in the quality of life [3].

The choice of LUTS treatment for BPH depends on clinical findings, treatment efficacy, treatment preferences, and patient expectations in terms of rate of onset, efficacy, side effects, quality of life, and disease progression. The first-choice therapy for male patients with LUTS and BPH is behavior modification, either with or without alpha-blockers. Conservative treatment (i.e., distraction techniques and bladder retraining) followed by anticholinergics is recommended when OAB symptoms persist after the administration of alpha-blockers [4]. Anticholinergics are the second-choice drug treatment for OAB symptoms.

Silodosin is highly selective for α1A-adrenoreceptors, which occur in the prostate, prostatic urethra, bladder base, and bladder neck. The inhibition of α1A-adrenoreceptors causes smooth muscle relaxation in these tissues and reduces bladder output obstruction. The effect is to alleviate lower urinary tract symptoms caused by BPH [5,6,7].

According to the Cochrane Urology Group, the method of the first treatment for overactive bladder in men with BPH and LUTS is pelvic floor muscle training (PFMT). PFMT is a method based on scientific evidence. It is defined as the repeated contraction and relaxation of the pelvic floor muscles. In symptoms of OAB in men, it is important to manage the suppression of urgency through the contraction of the rectal muscles [8]. Not enough studies on the combination of silodosin and PFMT in men with BPH and OAB have been published in the literature. To ensure the maximum effect of the intervention, it is necessary to constantly modify the exercise programs and update the knowledge in the relevant guidelines. It is important that the PFMT is interesting and that patients are motivated to complete the training program.

Anti-cholinergics are the basic drug treatment for OAB symptoms as a secondary choice. This is evidenced by several clinical trials that have confirmed the subsequent relief of OAB in patients with BPH after initial treatment with alpha-blockers [7,9,10]. Matsukawa et al. [11] compared the efficacy of combination therapy with silodosin and mirabegron 50 mg or fesoterodine 4 mg in patients with persistent OAB symptoms. At three months, there was a more significant reduction in OAB in the fesoterodine group than in the mirabegron group.

In the literature, there is a lack of published studies that examine a combination of silodosin and PFMT in men with OAB.

Silva [8] in a Cochrane system review of studies from various databases (CENTRAL, MEDLINE, Embase, Web of Science, LILACS, ClinicalTrials.gov, and WHO ICTRP), described information from studies on the effect of physical activity in men with BPH and LUTS. He included six randomized studies with 652 men over the age of 40 with mild to severe LUTS symptoms. The interventions were pelvic floor muscle training and Tai-chi. However, the quality of these studies was low, and the results were insufficient in terms of training the pelvic floor muscles for LUTS symptoms. The authors of the study point to the need for other high-quality randomized studies.

### 1.1. Primary Goal

The primary goal of this trial is to assess the efficacy of combination therapy with silodosin and pelvic floor muscle exercise (PFMT) versus silodosin treatment in men with BPH and OAB, according to the change in the number of voidings and intensity of urgencies over 24 h using a micturition diary, over 12 weeks of treatment.

### 1.2. Secondary Goals

The secondary goals are to assess changes in lower urinary tract symptoms (LUTS) using the International Prostate Symptom Score (IPSS), changes in incontinence quality of life using the Overactive Bladder Questionnaire (OAB-q), changes in the patient’s global impression of improvement, and the incidence of adverse events in the combined silodosin and pelvic floor muscle training compared to silodosin treatment alone over 12 weeks of treatment.

## 2. Materials and Methods

### 2.1. Study Design

The Silodosing study is designed as a randomized controlled intervention and parallel phase IV study. The Silodosing study will compare the effect of silodosin treatment and combination treatment with silodosin and PFMT in men with BPH and OAB treated with silodosin. The hypothesis is that after 12 weeks of combination therapy, the experimental group will have significantly fewer episodes in the number of voidings and intensity of urgencies over 24 h, a reduction in the prostate symptom score, and improvements in incontinence quality of life and patient global impression of improvement. The protocol was approved by the Ethics Committee of the University Hospital in Martin, Slovakia, Comenius University, number 09072020. All important protocol modifications will be approved by the same ethics committee. Informed consent will be obtained from trial participants by the local clinic of urology. Personal information about potential and enrolled participants will be collected, shared, and protected in concordance with Slovakian law.

### 2.2. Allocation

The Silodosing study will be performed with 45 urological outpatient clinics at the national level. It will be carried out in two parallel groups: the experimental group (E) and the control group (C). It will be a randomized controlled study with a 1:1 allocation ratio. Computer-generated sequences will be used for the allocation process by a researcher who will not participate in the study. The computer will generate an even and odd patient number. Odd-numbered patients will be given silodosin monotherapy at a dose of 8 mg once daily for 12 weeks, while even-numbered patients will receive combination treatment with silodosin at a dose of 8 mg once a day and pelvic floor muscle exercises at the same time. The generated numbers will be placed in sealed envelopes. Each envelope will contain the code of the experimental (E) group and control (C) group. The blinding will prevent deliberate selection (Table 1).

### 2.3. Sample Size Estimation

We used an estimate made by sampling the number of probands based on a power of test using 0.80 and alpha 0.05 (type I error). Using a statistical program, we determined the calculation of the number of probands; there will be 63 in the experimental group and 63 in the control group. We expect a 20% loss, so we will include at least 158 probands. According to the sample selection, we expect a 20% decrease in incidence after the intervention.

### 2.4. Inclusion Criteria

Willing to provide written informed consent. Men over 50 years with lower urinary tract symptoms, overactive bladder, and benign prostatic hyperplasia. Persistence of overactive bladder despite 4 weeks of silodosin treatment. Symptoms of overactive bladder (urinary frequency and urgency with or without urinary incontinence) for ≥3 months prior to Visit 1. Willing and able to complete the 3-day voiding diary and questionnaires. International Prostate Symptom Score (IPSS) score ≥8. Experience an average of 8 or more micturitions per day over the 3-day diary period. Experience an average of 2 episodes of urgency per day (grade 3 or 4) over the 3-day diary period.

### 2.5. Exclusion Criteria

Post-void residual volume (PVR) >200 mL. Evidence of urinary tract infection and hematuria. Use of anti-cholinergics or beta 3 mimetics within 4 weeks prior to Visit 1 and during the study. Oncological diseases of the lower urinary tract and prostate. Neurogenic bladder. Urethral strictures and bladder neck stenosis. Urolithiasis. Diabetes mellitus. Previous surgery of lower urinary tract. Stress urinary incontinence. Intermittent catheterization. Chronic urinary tract infection. Previous Botox treatment in the last 12 months. Chronic electrostimulation treatment of OAB in the last 12 months. Patient began or has changed a bladder training program or pelvic floor exercises less than 90 days before enrolment (T0). Cognitive deficits and dementia. Man has participated in an interventional trial within 30 days prior to enrolment (T0). Total daily urine production over 2500 mL according to voiding diary.

### 2.6. Procedures

#### 2.6.1. Experimental and Control Groups

Continuation of silodosin treatment at a dose of 8 mg daily for 12 weeks.

#### 2.6.2. Experimental Group

Peroral treatment with silodosin at a dose of 8 mg daily. 

Behavioral intervention: pelvic floor muscle training (PFMT) with urgency-suppression techniques.

The urologist will evaluate the strength of the rectal muscles before and after intervention by digital rectal examination (DRE). PFMT intervention will be performed by a trained physiotherapist, who will explain the correct exercise technique. Muscle strength and intra-anal endurance will be tested. PFMT will be conducted 5 days a week and supervised by a physiotherapist once a week. If the patient is able to perform PF muscle contractions correctly, they will be allowed to continue with the exercises at home (Table 2).

1. Education about proper urination training in the period without pathological urgency;
2. Education in urgency-suppression techniques:
  Urgency-suppression techniques are methods/maneuvers that are used to decrease the feeling of urgency. They include (but are not limited to): distraction; PFM contraction; perineal pressure such as sitting on a hard chair; relaxation; breathing; toe-curling; and plantar flexion of the ankle [12];
3. Education on the principle and effect of PFMT;
4. Education about proper ergonomics during everyday activities;
5. Education about the correct breathing stereotype;
6. Exercise about pelvic floor muscle awareness;
7. Exercises of varying intensity of the pelvic floor muscles in different positions;
8. Pelvic floor muscle relaxation exercises.

Exercise 5 times a week for 30 min a day, for 12 weeks.

### 2.7. Outcome Measures

#### 2.7.1. Primary Outcomes

Change in the number of voidings and intensity of urgencies over 24 h using a voiding diary. Voiding diary includes voided volume over 24 h (mL), the number of voidings per 24 h, voided volume during the day (mL), the number of voidings per day, voided volume during the night (mL), night-time frequency (nocturia), and mean voided volume per 24 h, during the day and during the night (mL) for 3 days, and the mean values were used.

The Patient Perception of Intensity of Urgency Scale (PPIUS) evaluates the severity of symptoms of an overactive bladder: 0 = no urgency, retaining urination for a very long time without fear of voiding; 1 = moderate urgency, able to hold urine as long as necessary without fear of voiding; 2 = moderate urgency, able to hold urination for a short while without fear of voiding; 3 = serious urgency, unable to hold urine; 4 = urgency urinary incontinence, urine leakage occurs before reaching the toilet. Reliability and validity are 0.95, and Spearman’s correlation is 0.89 [13].

The definition of an overactive bladder (OAB) is when patients have an urgency 1 or more times a day, voiding 8 or more times a day, and nocturia 2 or more times at night, with or without urgency urinary incontinence [1].

#### 2.7.2. Secondary Outcomes

##### The International Prostate Symptom Score (IPSS)

This is a 7-point scale for assessing the symptoms of benign prostatic hyperplasia (BPH). Seven questions focus on the symptoms of BPH. Rating: 0–7 mildly symptomatic, 8–19 moderately symptomatic, and 20–35 severely symptomatic. One question focuses on quality of life. On the scale, 0 = delighted, 1 = pleased, 2 = moderate symptoms, and 5 = severe symptoms. Cronbach’s alpha IPSS ≥ 0.60 and ≥0.79, respectively. The intra-class correlation coefficient was high (≥0.59) [14,15].

##### Overactive Bladder Questionnaire (OAB-q)

This questionnaire focuses on the symptoms of OAB incontinence in the last 4 weeks. It contains 6 questions with symptom scores (0 without symptoms, 100 most symptoms) and 13 questions that evaluate quality of life (100 best quality of life, 0 worst quality of life). Cronbach’s alpha OAB-q is 0.90 [16,17]. The Overactive Bladder Symptom Score (OABSS) has 7 questions and 5 answers. It is used to examine the symptoms of an overactive bladder. Total score is 0 = no symptoms, and 28 = severe symptoms. Spearman’s correlation coefficient was 0.84 [18].

##### Patient Global Impression of Improvement Score (PGI-I)

Condition of urination problems compared to the condition before a patient started treatment in the study. Scores: 1 = much better, 2 = quite better, 3 = a little better, 4 = no change, 5 = a little worse, 6 = a lot worse, and 7 = much worse [19].

##### Incidence of Adverse Events

Monitoring of treatment side effects and serious side effects. Very common side effects are ejaculatory disorders, including retrograde ejaculation and anejaculation. Common side effects are dizziness, orthostatic hypotension, nasal congestion, and diarrhea [20].

### 2.8. Adherence

Competence, cooperation, and security will be recorded during the recruitment and implementation of the study. The principal investigator, in collaboration with urologists, will register suitable patients from all databases in outpatient clinics and registries. The number of eligible patients from all enrolled patients will be recorded. During the intervention period, cooperation and therapeutic adherence to the exercise regimen and drug treatment, as well as treatment interruption, will be recorded in the diary. 

### 2.9. Statistical Analysis

Descriptive and inferential statistics will be used for data analysis. The unpaired t-test will be used to compare the experimental and control groups before training. Our data will have a normal distribution. Differences between control and experimental groups before and after the intervention will be evaluated by General Linear Model (GLM), Mixed Design ANOVA—repeated measurements with Greenhouse–Geisser correction. The significance level will be set at 95%, *p* < 0.05. Effect size (ES) will be calculated based on the Partial Eta Squared (η^2^). According to Cohen, the specification for ANOVA analysis of small, medium, and large effect size will be classified as η^2^: 0.00–0.003: no effect size; η^2^: 0.010–0.039: small effect size; η2: 0.060–0.110: medium effect size; η^2^: 0.140–0.200: large effect size. Calculations will be performed in IBM SPSS Statistics for Macintosh, Version 27.0 (IBM Corp., Armonk, NY, USA).

### 2.10. Monitoring

The study will be controlled by an independent person. The Commission will be the decisive authority of the study. The principal researcher—investigator J.S.—will be responsible for the organization of research activities and communication with patients, collaborators, and partners. The coinvestigator MH will manage central randomization, project and ethical standards, data collection, protection, data entry, data storage, and processing. The explanation of the examination and exercises in the given study will be achieved by 45 members of the research team.

## 3. Conclusions

The study protocol presents the starting points and design of a randomized, interventional, multicenter study monitoring the effect of combination therapy with silodosin and pelvic floor muscle training (PFMT) in comparison with silodosin treatment alone in men with BPH and OAB. The study may provide evidence of the efficacy of combined treatment for OAB and indicate an active approach for the treatment of OAB through exercise. The strength of the study is that it promotes a non-invasive and conservative treatment of strengthening pelvic floor muscles with the suppressive-urgency technique in addition to silodosin treatment. We will use standardized measuring tools: the voiding diary for the assessment of changes in the number and intensity of urgencies over 24 h [1], the Patient Perception of Intensity of Urgency Scale (PPIUS) [13,19,21], the International Prostate Symptom Score (IPSS) for the assessment of prostate symptoms [13,14], the Overactive Bladder Questionnaire (OAB-q) for assessing changes in incontinence quality of life [16,17], the Overactive Bladder Symptom Score (OABSS) to examine the symptoms of an overactive bladder [18], and the Patient Global Impression of Improvement score (PGI-I) to assess the condition of urinary problems compared to pre-treatment [22].

The strength of the study is its support of the innovative non-invasive conservative treatments of pelvic floor muscle training and medication with silodosin in patients with BPH and OAB.

The Methodology section describes the ranking criteria, method of inclusion in the study, and randomization procedure. The patients will be men aged over 50 with BPH and OAB. Within that demographic, the following parameters will be monitored: age of probands, education, height, weight, employment, smoking, alcohol, hypertension, lung diseases, nervous diseases, cardiovascular diseases, and other diseases.

The intervention in both groups will be oral treatment with silodosin at a daily dose of 8 mg; in the experimental group, pelvic floor muscle training (PFMT) with the suppressive urgency technique will be added. Interventions will last 12 weeks, with exercises five times a week for 30 min a day, under the supervision of a physiotherapist. Education will consist of education about proper urination training in the period without pathological urgency, education about conscious urgency suppression through the PFMT suppressive urgency technique, education on the principle and effect of PFMT, education about proper ergonomics during everyday activities, education about the correct breathing stereotype, and education regarding the correct understanding of exercises by physiotherapists in cooperation with a nurse.

The program includes exercises about pelvic floor muscle awareness, exercises of varying intensity of pelvic floor muscles in different positions, and pelvic floor muscle relaxation exercises. Subsequently, the exercise will be performed at home with a diary check.

Based on similar studies, we expect good cooperation, small losses of probands, and agreement with the study protocol. Patients who are assigned to silodosin only will be offered the opportunity to exercise after 12 weeks of treatment. We assume a 20% loss. We will consider the success of the treatment to be a decrease of more than 50% in remission.

## Figures and Tables

**Table 1:** Recruitment, intervention, and evaluation plan for the experimental and control groups in the Silodosing study.

Time Period	Enrolment (T0) before Treatment (T1)	After 12 Weeks of Treatment (T12)
Informed consent	x	
Enrolment	x	
Allocation	x	
Demographic data	x	
Voiding diary	x	x
The International Prostate Symptom Score (IPSS)	x	x
Overactive Bladder Questionnaire (OAB-q)	x	x
Overactive Bladder Symptom Score (OABSS)	x	x
The Patient Perception of Intensity of Urgency Scale (PPIUS)	x	x
Patient Global Impression of Improvement score (PGI-I)		x
Adverse events—harms	x	x
Adherence	x	x
Intervention for experimental group	x	x

**Table 2:** Exercise program for men with BPH and OAB.

	Description	Dosage
1	Education about proper urination training in the period without pathological urgency. Education about conscious urgency suppression through PFMT—the suppressive urgency technique. Education about healthy urge to urinate and pathological urge to urinate. Education on how to cancel a sudden, urgent urge to urinate: a. Tighten the rectal muscles and pull them as if inside the body and hold on until the urge to be in your current position is lifted; b. If you cannot keep your rectal muscles tightened, for 2 s, release them and download them again.	5 times
2	Education on the principle and effect of PFMT. Do not contract the sciatic muscles, abdominal muscles, and lower limbs instead of the rectal muscles. The principle of exercise: by compressing the muscles of the rectum and pulling them as if inside the body, you also affect the muscles around the urinary tract.	5 times
3	Education about proper ergonomics during everyday activities. Education about the correct breathing stereotype. When lying on your back or sitting, keep your chest in an exhaled position. With a breath to the sides of the abdomen, you activate the correct diaphragmatic breathing. With exhalation, pull the navel to the spine, activating the deep abdominal muscles.	5 times
4	Exercise Phase 1: Weeks 1 and 2 Exercise 1: Pelvic floor muscle awareness Exercise positions: Lying on the back—legs bent, lying on the abdomen, then sitting, standing. Exercise: Awareness of the rectal muscles. Inhale—imagine that you want to draw the rectal muscles inside the body and exhale and relax the rectal muscles, in the rhythm of normal breathing, repeat 10–20 times.	15 min a day
5	Exercise Phase 2: weeks 3–12 Exercise 2: Exercises to strengthen the pelvic floor muscles Exercise positions: Spine, abdomen, sitting, semi-sitting, standing, walking (medium strength and very strong rectal muscle contraction). Exercise: a. Tighten the rectal muscles and pull them into the body moderately for 10 s and then relax for 10 s. b. Tighten the rectal muscles and pull them into the body very hard for 5 s and then relax them for 5 s.	15 min a day
6	Exercise 3: Pelvic floor muscle relaxation exercises Exercise positions: Lying on your back, then sitting. Exercise: a. Tighten the rectal muscles moderately for 1 s and then relax for 10 s. b. Tighten the rectal muscles slightly for 5 s and then relax for 10 s.	15 min a day

## Data Availability

We have not received ethical approval to share raw clinical trial data. The data that will be collected will be available upon request to the author.

## Abbreviations

PFMT	Pelvic floor muscle training
PGI-I	Patient Global Impression of Improvement
OAB-q	Overactive Bladder Questionnaire
OABSS	Overactive Bladder Symptom Score
PPIUS	The Patient Perception of Intensity of Urgency Scale
IPSS	The International Prostate Symptom Score
GLM	General linear model
ANOVA	Analysis of variance
